# Host-Parasite Interaction between Parasitic Cymothoid *Ceratothoa oestroides* and Its Host, Farmed European Sea Bass (*Dicentrarchus labrax*)

**DOI:** 10.3390/pathogens9030230

**Published:** 2020-03-20

**Authors:** Ivona Mladineo, Jerko Hrabar, Olja Vidjak, Ivana Bočina, Slavica Čolak, Pantelis Katharios, Maria Chiara Cascarano, Kleoniki Keklikoglou, Donatella Volpatti, Paola Beraldo

**Affiliations:** 1 Institute of Oceanography and Fisheries, 21000 Split, Croatia; hrabar@izor.hr (J.H.); vidjak@izor.hr (O.V.); 2 Faculty of Science, University of Split, 21000 Split, Croatia; bocina@pmfst.hr; 3 Cromaris doo, 23000 Zadar, Croatia; slavica.colak@cromaris.hr; 4 Hellenic Center for Marine Research, Heraklion, 712 01 Crete, Greece; katharios@hcmr.gr (P.K.); mariachiaracascarano@gmail.com (M.C.C.); keklikoglou@hcmr.gr (K.K.); 5 Section of Animal and Veterinary Sciences, Department of Agricultural, Food, Environmental and Animal Sciences University of Udine, 33100 Udine, Italy; donatella.volpatti@uniud.it (D.V.); paola.beraldo@uniud.it (P.B.)

**Keywords:** attachment, *Ceratothoa oestroides*, European sea bass, feeding, host-parasite interaction

## Abstract

Parasitic isopod *Ceratothoa oestroides* (Cymothoidea, Isopoda) is a common and generalist buccal cavity-dweller in marine fish, recognised for its detrimental effect in fingerling and juvenile farmed European sea bass (*Dicentrarchus labrax*). Although distributed throughout the Mediterranean, the isopod provokes acute outbreaks mainly limited to particular endemic areas in Croatia (Adriatic Sea) and Greece (Aegean Sea). While numerous studies have previously evidenced its gross effect on farmed fish (i.e. decreased condition index, slower growth rate, lethargy and mortality), details on the host-parasite interaction are still lacking. Therefore, using a multimethodological approach, we closely examined the structure and appearance of isopod body parts acting in the attachment and feeding (stereomicroscopy, scanning and transmission electron microscopy), and the extent of host tissues damage (histology, immunohistochemistry, micro-computational tomography) induced by parasitation. Interestingly, while hematophagous nature of the parasite has been previously postulated we found no unambiguous data to support this; we observed host tissues fragmentation and extensive hyperplasia at the parasitation site, and no structures indicative of heme detoxifying mechanisms in the parasite gut, or other traces of a blood meal. The bacterial biofilm covering *C. oestroides* mouthparts and pereopods suggests that the isopod may play a role in conveying secondary pathogens to the infected host, or alternatively, it serves the parasite in normal interaction with its environment.

## 1. Introduction

Family Cymothoidae encompasses obligate, mostly marine isopod parasites, and forms the superfamily Cymothooidea Wägele, 1989 with families Corallanidae, Aegidae and Tridentellidae [1]. Cymothoidae are typically parasitic in teleost fish, although they also infect Chondrichthyes, jellyfish, cephalopods, crustaceans and amphibians. They exhibit a wide diversity of strategies for the niche colonisation, being able to attach on the external surfaces or gills, as well as to burrow flesh or dwell in the buccal cavity. The rest of families has adapted to one of many possible host interactions; from commensalism to micropredation [2]. 

*Ceratothoa oestroides* (Cymothoidea, Isopoda) is a generalist species within Cymothoidae family, being recorded in an inconclusive number of different fish families: Sparidae (bogue *Boops boops*, annular sea bream *Diplodus annularis*, common pandora *Pagellus erythrinus*, picarel *Spicara smaris*, gilthead sea bream *Sparus aurata*), Carangidae (Mediterranean horse mackerel *Trachurus mediterraneus*), Clupeidae (European pilchard *Sardina pilchardus*), Maenidae, Scorpenidae (small red scorpionfish *Scoarpena notata*, black scorpionfish *Scoarpena porcus*) and Mugilidae (golden grey mullet *Liza aurata*) [3,4]. Its direct life cycle includes mating of the paired adults settled in fish buccal cavity, embryonic development to pullus I and II stage, release of infective and swimming stage or pullus II (manca), its attachment to the novel hosts and protandric maturation. The latter encompasses puberty period of male, transitory stage, puberty period of female and finally maturation of the female [5,6]. 

In aquaculture, *C. oestroides* infects mainly European sea bass (*Dicentrarchus labrax*) [7], and to a lesser extent gilthead sea bream (*Sparus aurata*) [8]. With the recent expansion of meagre (*Argyrosomus regius*) farming in the Adriatic Sea, the isopod has been also recorded in meagre fry (6.53 ± 1.26 g) after the transfer in sea cages [9]. Susceptibility of reared fish seems to be age-related, since pulli II attached to small fry or fingerlings inflict the most damage, causing abrupt cachexia, anaemia, terminal emaciation and 5–20% mortalities [6]. Although some authors observed severe ulcers, extensive granulomatous lesions that cause blindness or the total loss of the eyeball [10], fish usually suffer mortalities after chronic emaciation without displaying specific macroscopic lesions. Larger fish infected with adult parasites do not show serious clinical signs, and lesions are mainly localized at the upper and lower jaws and the tongue [10] or observed as deformities of the buccal ventral part [9]. However, sea bass harvested prior the transport to retailer need to be checked and “de-loused” manually at the sorting facility, as the finding of large isopods in the fish buccal cavity is fastidious for consumers and does not comply with EU regulatory rules on the hygiene of food of animal origin [11]. Growth performances of infected fish can be reduced up to the 20% [12,13], or impaired by post-haemorrhagic anaemia [14]. Horton and Okamura [13] observed growth and length reduction in a pre-commercial size sea bass (291-293 days post hatching) amounting up to 20.1% (14 g) and 7.1% (12.63 mm), respectively. However, growth retardation seems to be affected by season, as infected cultured meagre monitored over six months of grow-out period showed the greatest reduction in length and weight in March (e.g. 33% and 74%, respectively compared to uninfected fish), probably related to an unfeasible demand for a metabolism increment in infected fish, induced by sharp increase of seawater temperature after the winter [9].

In order to design appropriate prophylactic and therapeutic strategies for the mitigation of *C. oestroides* infection in aquacultured fish, it is necessary to understand parasite’s biology and epidemiology, its interaction with the host, as well as the underlaying environmental conditions that help shaping it. In that context, herein we described a range of unknown traits of adult *C. oestroides* parasitation in the buccal cavity of European sea bass fingerlings using a multimethodological approach: i) histology and immunohistochemistry to assess tissues damage and infer the feeding strategy of the isopod; ii) 2 and 3D visualisation using micro-computational tomography (µ-CT) to gain robust data on parasite attachment strategy; iii) stereomicroscopy to describe ontogeny of mouthparts in *C. oestroides* intramarsupial embryonic stages; iv) scanning electron microscopy (SEM) of parasite’s appendages in direct contact with host tissues to support µ-CT data; and v) transmission electron microscopy (TEM) of the parasite’s gut to expand fundamental knowledge on its ultrastructure.

## 2. Results

### 2.1. Histology (Figure 1) and Immunohistochemistry (Figure 2)

Paired male and female were settled at the floor of oro-pharyngeal cavity (buccal cavity and pharynx) of infected European sea bass; female mostly logged centrally in the buccal cavity, and the male in the pharynx, the latter attached right or left laterally to gill rakers. Only large females were observed occupying also the pharynx.

**Figure 1:**
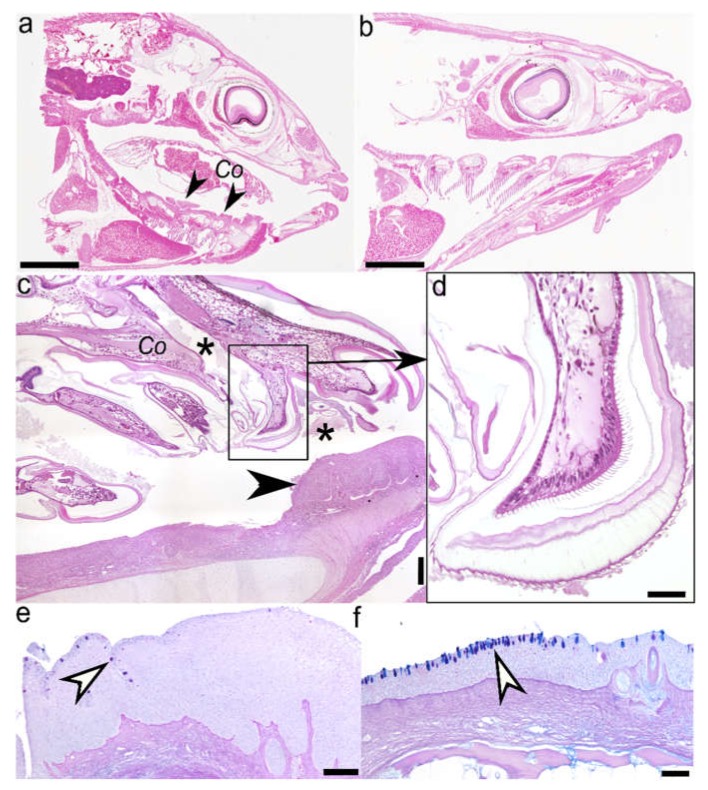
Adult female *Ceratothoa oestroides* parasitising the European sea bass (*Dicentrarchus labrax*) buccal cavity: (**a**) Longitudinal section through buccal cavity of infected fish. *Co*: *Ceratothoa oestroides*; black arrowhead: hyperplastic epithelium (scale bar = 5 mm); (**b**) Longitudinal section through buccal cavity of uninfected fish (scale bar = 5 mm); (**c**) Larger view of the anterior third of isopod’s body. Asterisks: fragmented and ingested host tissues; black arrow: magnification of the selected inset (scale bar = 200 µm); (**d**) Increased magnification of the isopod’s mandible (scale bar = 50 µm); (**e**) Stratified squamose epithelium of the tongue showing hyperplasia and reduction in mucous cells number in infected fish. White arrowheads: mucous gland cells (scale bar = 100 µm); and (**f)** uninfected fish. White arrowheads: mucous gland cells (scale bar = 100 µm.) H&E staining.

In general, infected buccal cavity, especially the tongue, showed a regressive appearance and seemed to be expanding in ventral area in respect to that in parasite-free fish (Figure 1a,b). However, although parasite body parts caused disruption and stunted growth of gill rakers with marked thickening over the inner edges of the gill rakers and branchial arches, no healing with consequent reparation by connective tissue was observed. Gill rakers damage involved also hard tissues, eliciting alteration of the rakers shape. Only the epithelium of oro-pharyngeal cavity showed a slight hyperplasia, mainly in the buccal cavity (Figure 1c).

Microscopically, lesions induced by parasite settlement showed a chronic inflammatory character, and included marked hyperplasia, cell desquamation, mixed leukocytic infiltration and decrease in mucous cell number. Lesions resulted from three main sources of trauma effected by parasitation; (i) mouthparts chawing (Figure 1d) and pointed ungulae of the pereopods inducing mechanical perforation of oro-pharyngeal epithelium; (ii) pleopods continuous beating that enables parasite breathing; and (iii) the whole parasite body that exerts pressure on oral cavity epithelium.

Diffuse and marked hyperplasia of squamous stratified epithelium is observed both in the tongue (buccal cavity), and gill arches and rakers (pharynx). Within this hyperplastic area, numerous cells undergoing mitosis are observed. The thickness of the tongue epithelium and gill arches is approximately four times greater than that recorded in uninfected fish epithelium (0.2 vs 0.05 mm), but no thickening of underlaying connective tissue layer is observed. Furthermore, mucous cells scattered within the epithelium of oropharyngeal cavity are reduced approximately by a third in their number, in respect to uninfected fish (Figure 1e,f). Cell desquamation is also marked in tongue, especially at the site of focal ulcerations provoked by pereopod ungulae.

The inflammatory response is characterised by an abundant leukocyte infiltrate in the connective tissue, in particular under the basal membrane, consisting predominantly of lymphocytes, a moderate number of mast cells and very few eosinophilic granulocytes.

Interestingly, no haemorrhaging is observed in the area of ungulae attachment, nor at the contact of mouthparts with the epithelium. In contrast, histological sections show that epithelium fragments are present in close proximity of mandibles (Figure 1c).

Out of seven cell markers tested by immunohistochemistry (ESB IgM, iNOS, PCNA, Cytokeratin, CRP, Cox-2 and CD83), ESB IgM, Cytokeratin (data not shown), Cox-2 and PCNA positively reacted with antigens expressed in tissues. Reactivity of antibodies developed against mammalian antigens does not always allow to positively label antigens expressed by fish tissues, but still some of those evolutionarily highly conserved proteins can give rise to cross reactivity. 

**Figure 2:**
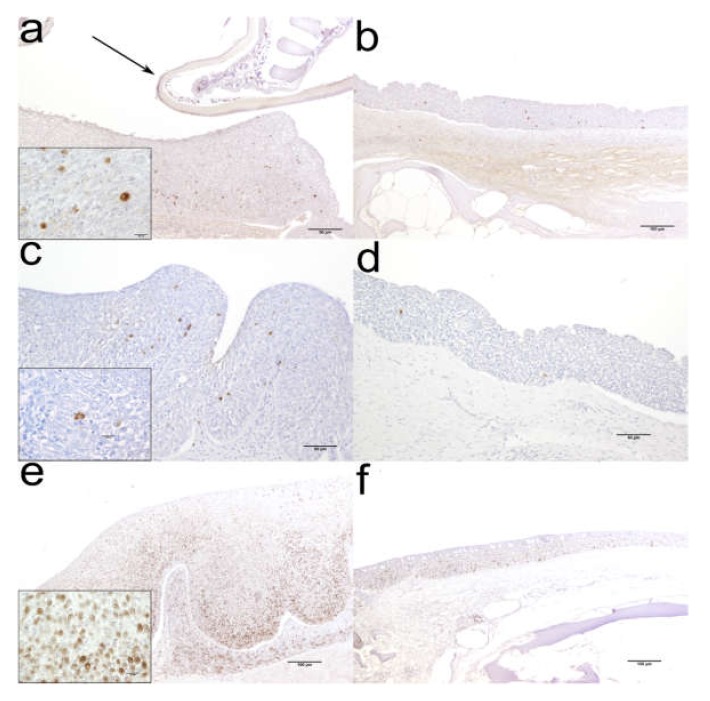
Representative micrographs of different immunolabelling in the farmed European sea bass (*Dicentrarchus labrax*) oral mucosa: (**a**) Moderate number of IgM^+^ cells in infected fish, compared to weak expression in (**b**) uninfected fish. Inset: higher magnification of IgM^+^ cells; arrow: isopod’s pereopod; (**c**) Weak number of Cox-2^+^ cells in infected fish, compared to very low number in (**d**) uninfected fish. Insert: higher magnification of Cox-2^+^ cells; (**e**) Very high number of PCNA^+^ cells in infected fish, compared to moderate number in (**f**) uninfected fish. Insert: higher magnification of PCNA^+^ cells.

Anti-ESB IgM labelled the cytoplasm of cells in deeper layers of the hyperplastic squamous stratified epithelium of the oral cavity, most likely B-cells, plasmablasts and/or plasma cells. Even though IgM^+^ cells were also present in uninfected oral mucosa, their number was considerably increased in infected fish (Figure 2a,b). Cox-2 molecule was expressed in the cytoplasm of sparse cells, mainly in basal and to a lesser degree in superficial mucosal layer, but with feeble differences between uninfected and infected fish (Figure 2c,d). PCNA^+^ cells were markedly increased in number both in squamous epithelium mucosa and lamina propria of infected fish. The positive nuclear signal was especially abundant in basal mucosal layer, then in the intermedial layer, although it reached superficial layer as well. Signal in the lamina propria was more enhanced in deeper layer, compared to superficial layer (Figure 2e,f).

No staining was observed in sections where specific primary antibody was replaced by the dilution buffer (control by omission), confirming specificity of the reactions (images not shown). 

### 2.2. Micro-computational Tomography (µ-CT) (Figure 3)

**Figure 3:**
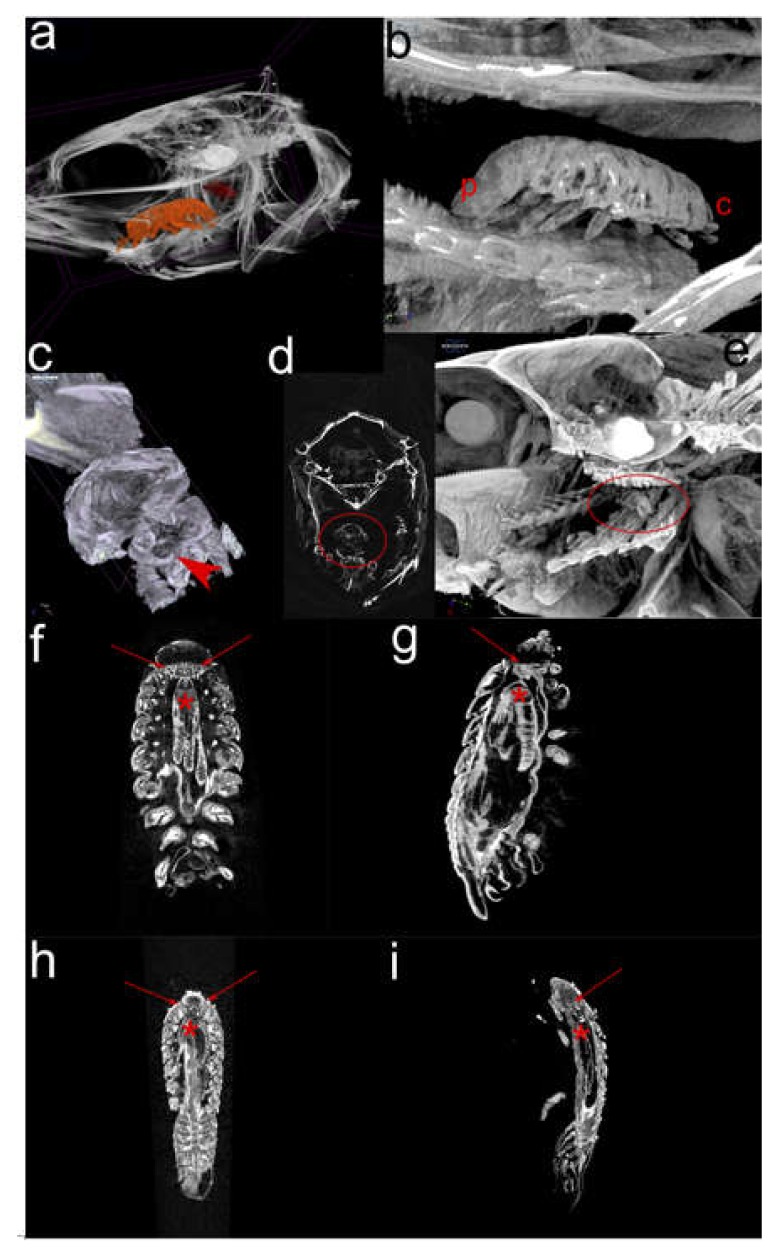
Selected renderings of 2 and 3D micro-computational tomography (µ-CT) of adult female and male *Ceratothoa oestroides* parasitising European sea bass (*Dicentrarchus labrax*) buccal cavity: (**a**) *In situ* 3D visualisation of female (orange) and male (red) positions; (**b**) Magnified 3D rendering of female showing attachment of the pereopods (p: pleotelson; c: cephalon); (**c**) 3D rendering of posterior paired pereopods piercing deep into lower limbs of gill arches (arrowhead); (**d**) Frontal 2D visualisation of female showing no direct contact between her mouthparts and host tissues (encircled); (**e**) Lateral 3D visualisation of male position (encircled); (**f**) Longitudinal 2D visualisation of female showing gland-like structures in the cephalon region (arrows) and mid-gut diverticula (asterisk), and (**g**) same, but in the lateral plane; (**h**) Longitudinal 2D visualisation of male showing no gland-like structures in the cephalon region (arrows) and mid-gut diverticula (asterisk), and (**i**) same, but in the lateral plane. Note that the software for figure construction dose not enable scale bar insertion, therefore, refer to other figures for the dimensions.

In selected *C. oestroides*-infected sea bass (Figure 3a), three orally-directed pereopod pairs of female parasite deeply pierced isthmus part of the lower limb of gill arches, while the rest of aborally-directed pereopods pierced dividing lower limbs of gill arches (Figure 3b,c); all pereopods following their anatomical distribution. Female mouthparts were not observed piercing host tissues (Figure 3d). The male was laterally logged on the right side, transversally overpassing three gill arches (lower limbs), with no direct contact with the female (Figure 3a,e).

Longitudinal 2D renderings of female (Figure 3f) showed that bilaterally, between a short oesophagus and cuboid ventral ampule, two gland-like structures are present that are missing in male (Figure 3h). The appearance of six aborally extending midgut diverticula, showed more dense structure in females (Figure 3g), compared to more vacuolated structure in males (Figure 3i). 

### 2.3. Description of C. oestroides Intramarsupial Embryonic Stages and Mouthparts (Figure 4)

**Figure 4:**
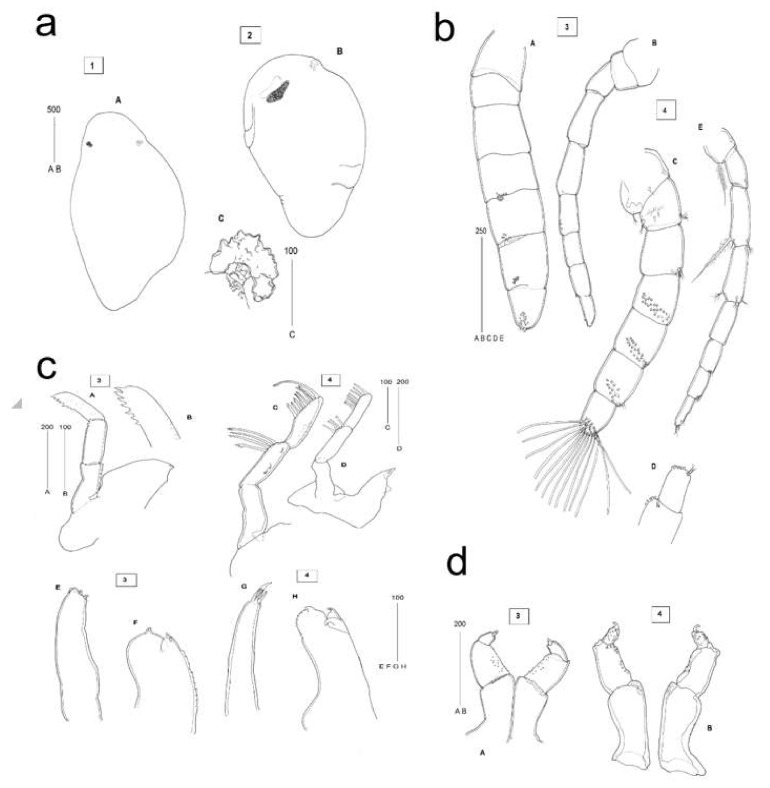
Intramarsupial embryonic stages of *C. oestroides*: (**a**) Habitus (A, B) of morphotypes 1 and 2 of early pullus I and embryonic dorsal organ (C) of morphotype 2; (**b**) Head appendages in older pullus I - antennule (A) and antenna (B) of morphotype 3, and antennule (C) with detail of terminal article (D) and antenna (E) of morphotype 4; (**c**) Mouthparts in older pullus I - mandible (A) with tip of mandibular palp (B) of morphotype 3; mandible (C, D) of morphotype 4; maxillule (E, G) of morphotypes 3 and 4, and maxilla (F, H) of morphotypes 3 and 4; (**d**) Mouthparts of older pullus I - maxillipeds (A, B) of morphotypes 3 and 4. All scales are in micrometres.

Intramarsupial embryonic stage 2: Body elongated, with distinct head-end and early buds of appendages; small irregular darkly pigmented area situated at the presumed eye location; rudiment of the embryonic dorsal organ visible posterior to the eye region along dorsal midline (Figure 4a-A).

Intramarsupial embrionic stage 3 (pre-hatch 1): Body elongated, with appendages visible as small rounded projections along ventral side; posterior end with traces of somites formation; eyes more conspicuous than in the earlier stage (Figure 4a-B); embryonic dorsal organ present and conspicuous, with cells arranged in a circular patch invaginated at the centre (Figure 4a-C).

Head appendages, morphotype 3 (pullus I, pre-manca): Antennule stout, subequal in length to antenna, comprising 7 articles. Peduncular articles 1-3 distinctly articulated, subequal in width to flagellar articles; terminal flagellar article incompletely separated from the subterminal; marks of future pattern of sensory setae visible on ventral side of articles 4-7, other articles non-setose (Figure 4b-A). Antenna slenderer than antennule, comprising 9 articles, with terminal article incompletely articulated. Peduncular articles 1 and 2 broader than the rest, articles 4 and 5 elongated. Rudimentary setae present on flagellar articles 8 and 9 (Figure 4b-B). Mandible with prominent palp, composed of 3 elongated articles. Palpar articles 1 and 2 without setae, article 3 with several rudimentary serrate setae. Mandibullar process ending in small incisor (Figure 4c-A,c-B). Maxillule simple, with 4 small blunt apical spines (Figure 4c-E). Maxilla bilobed, covered with pectinate scales along inner margin. Lateral lobe bearing 1, and medial lobe bearing 2 small stout spines (Figure 4c-F). Maxilliped 3-segmented, palp article 3 with 2 small uneven apical spines (Figure 4d-A).

Head appendages, morphotype 4 (pullus I, pre-manca): Antennule stout, about as long as antenna, with all 7 articles distinctly articulated. Peduncular article 1 more expanded than in earlier stage. All articles setose; short plumose setae located on peduncular articles 1 (3 setae), 2 (7 setae) and 3 (4 setae). Flagellar articles 6 and 7 each with 2 simple small setae, articles 4-7 each adorned with more than 10 long sensory setae (Figure 4b-C,b-D). Antenna less robust than antennule, comprising 8 articles. Setation present on all articles, except on article 5. Plumose setae longer than segment present on peduncular articles 1 and 3, other setae short (Figure 4b-E). Mandible with distinctly 3-segmented prominent palp, longer than mandible. Palpar article 2 with 4 long subdistally serrated setae and article 3 with 7 shorter serrated setae and 1 long recurved terminal seta arising from the distolateral margin (Figure 4c-C,c-D). Mandibular incisor present. Maxillule simple, more styliform than in earlier stage, with 4 apical spines; the longest spine slightly recurved (Figure 4c-G). Maxilla bilobed, with 2 recurved spines on medial, and 1 recurved spine on lateral lobe, scaly along the inner margin (Figure 4c-H). Maxilliped comprised of 3 articles, articles 2 and 3 covered in pectinate setae. Article 3 short and ending in 2 recurved apical spines (Figure 4d-B).

Head appendages, morphotype 4 (pullus I, pre-manca): Antennule stout, about as long as antenna, with all 7 articles distinctly articulated. Peduncular article 1 more expanded than in earlier stage. All articles setose; short plumose setae located on peduncular articles 1 (3 setae), 2 (7 setae) and 3 (4 setae). Flagellar articles 6 and 7 each with 2 simple small setae, articles 4-7 each adorned with more than 10 long sensory setae (Figure 4b-C,b-D). Antenna less robust than antennule, comprising 8 articles. Setation present on all articles, except on article 5. Plumose setae longer than segment present on peduncular articles 1 and 3, other setae short (Figure 4b-E). Mandible with distinctly 3-segmented prominent palp, longer than mandible. Palpar article 2 with 4 long subdistally serrated setae and article 3 with 7 shorter serrated setae and 1 long recurved terminal seta arising from the distolateral margin (Figure 4c-C,c-D). Mandibular incisor present. Maxillule simple, more styliform than in earlier stage, with 4 apical spines; the longest spine slightly recurved (Figure 4c-G). Maxilla bilobed, with 2 recurved spines on medial, and 1 recurved spine on lateral lobe, scaly along the inner margin (Figure 4c-H). Maxilliped comprised of 3 articles, articles 2 and 3 covered in pectinate setae. Article 3 short and ending in 2 recurved apical spines (Figure 4d-B).

### 2.4. Scanning Electron Microscopy (SEM) of the C. oestroides Female (Figure 5)

**Figure 5:**
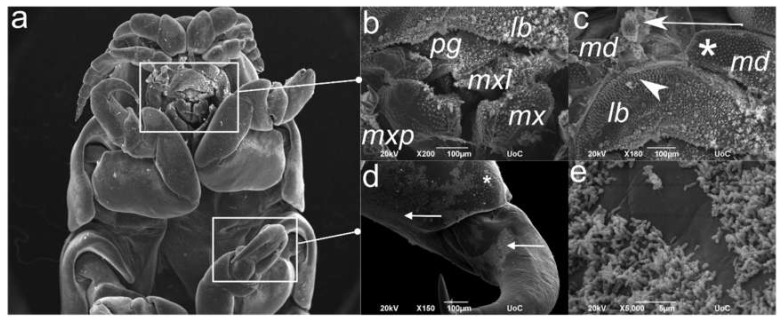
Representative scanning electron microscopy (SEM) renderings of female *C. oestroides* body parts in direct contact with host tissues: (**a**) Orientational ventral view with white rectangles focusing on subsequently magnified details; (**b**) Mouthparts (lb: labrum; mxl: maxillula; mx: maxilla; and mxp: maxilliped; pg: paragnath) covered with plumose spines; (**c**) Labrum (lb) covered with elongated scales with thorny processi (arrowhead) and mandibles (md) covered with triangular scales (asterisk). Note the host tissues fragments (arrow); (**d**) Distal pereopod parts covered with triangular spines (asterisk) and bacterial biofilm (arrows); (**e**) Higher magnification of bacterial biofilm on the pereopod ungula.

The morphological appearance of whole parasite body was beyond the scope of this study, and herein we focused on body parts (Figure 5a) in direct contact with host tissues, e.g. the mouthparts (Figure 5b) and distal pereopod parts (Figure 5d). The former are covered with plumose spines and elongated scales with thorny processes of varying length and coverage; most abundant on the frontal labrum surface (Figure 5c). In addition, mandibles and especially distal pereopod part up to ungulae, are covered with bacterial biofilm (Figure 5d,e).

### 2.5. Transmission Electron Microscopy (TEM) of the C. oestroides Female (Figure 6)

**Figure 6:**
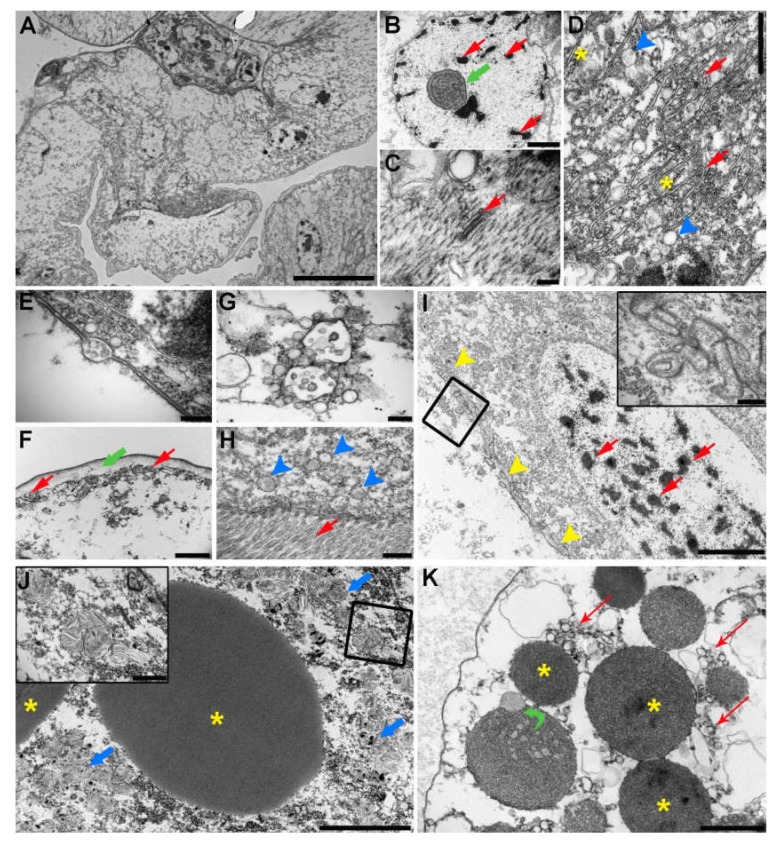
Representative transmission electron micrographs of *C. oestroides* intestine (a-g) and hepatopancreas (h-k): (**a**) Overview of single-layered intestinal wall composed of columnar epithelial cells with large nuclei (scale bar = 20 μm); (**b**) Large, euchromatic nucleus with small projections, prominent nucleolus (green arrow) and several small patches of heterochromatin (red arrows) (scale bar = 2 μm); (**c**) Numerous short filaments in cytoplasm of myocytes with a single Golgi apparatus (red arrow) (scale bar = 200 nm); (**d**) Apical portion of intestinal cell with numerous membranes of endoplasmic reticulum (red arrows), differently sized vesicles (blue arrowheads) and numerous free ribosomes (asterisk) (scale bar = 1 μm); (**e**) Transport of multivesicular body over cell membrane (scale bar = 200 μm); (**f**) Numerous short infoldings of basal cell membrane (red arrows) and thick basement membrane (green arrow) with thin electron-dense and thick electron-lucent layer (scale bar = 2 μm); (**g**) Two multivesicular bodies surrounded by numerous small vesicles in the basal portion of intestinal cell (scale bar = 200 nm); (**h**) Apical membrane of hepatopancreatic cell with microvili (red arrow) and several small vesicles adjacent to cell membrane (blue arrowheads) (scale bar = 500 nm); (**i**) Overview of hepatopancreatic cell with numerous infoldings of lateral cell membrane (yellow arrowheads) and large nucleus containing abundant heterochromatin (red arrows), inset: detail of membrane infoldings marked with black rectangle (scale bar = 5 μm, insert = 500 nm); (**j**) Large lipid droplets (asterisk) in cytoplasm of hepatopancreatic cells surrounded by numerous vesicles with likely proteinaceous content (blue arrows), inset = detail of vesicles marked with black rectangle (scale bar = 5 μm, insert = 1 μm); (**k**) Several electron-dense vesicles in cytoplasm of hepatopancreatic cell with granulated content (asterisk), indicative of peroxisomes, and surround by many small vesicles (red arrows). Note the fusion of peroxisome with a vesicle (green curved arrow) (scale bar = 1 μm).

The intestine of *C. oeastroides* is formed by a monolayer of columnar enterocytes with excentric and large nuclei (Figure 6a). The nuclei have prominent nucleoli with abundant electron-lucent euchromatin and several small patches of highly electron-dense heterochromatin (Figure 6b). Occasionally, cytoplasm of some cells is seen interspersed by numerous filaments between which different vesicles and small Golgi apparatuses can be seen (Figure 6c). Towards the apical portion, cells contain numerous membranes of endoplasmic reticulum, between which numerous vesicles and free ribosomes can be observed, resulting in more electron-dense appearance of this part of cells (Figure 6d). Occasionally, vesicular transport between cells is seen in the apical parts of the cells (Figure 6e). At the basal part, cells are connected to a thick basement membrane, composed of thin electron-dense and thick electron-lucent layer (Figure 6f). Cell membrane in this part has numerous invaginations (Figure 6f) and cytoplasm contains notable number of multivesicular bodies (MVBs) surrounded by numerous small vesicles (Figure 6g). Noteworthy, no particles of ingested food could be seen in the intestinal lumen. 

Apical membrane of cells of hepatopancreas is lined by densely packed microvilli. Below the membrane numerous small vesicles are present (Figure 6h). Large, ovoid nuclei are present in the basal portion and contain abundant euchromatin with more numerous patches of heterochromatin and of apparently lower electron density compared to intestinal cells (Figure 6i). Lateral membranes of hepatopancreas cells form numerous elaborate invaginations and small vesicles can be seen adjacent to the membranes (Figure 6i and Figure 6i inset). Numerous cells of hepatopancreas contain large, electron-dense lipid droplets (Figure 6j). These lipid droplets are often surrounded by many differently sized vesicles containing electron-lucent, likely proteinaceous content (Figure 6j inset). In a number of cells of hepatopancreas several differently sized vesicles of same electron density as lipid droplets can be seen, however with coarsely granulated appearance indicative of peroxisomes (Figure 6k). Interestingly, no crystalline inclusions indicative of hemozoin can be seen.

## 3. Discussion

Combining different microscopy techniques, we assessed interaction and effect of the isopod *Ceratothoa oestroides* on juvenile farmed European sea bass.

Observations of the mouthparts appearance in adult female and developing intramarsupial stages, gut ultrastructure of the former, as well as tissues alterations at the attachment site, give no unequivocal evidence that isopod has hemophagous nature. The gross morphology of mouthparts of early pulli is in general concordance with that of adults, presumably indicative of similarity in the feeding mode. The armature elements on maxillule, maxilla and maxillipeds consist of more or less recurved spines, suitable for the host tissue piercing and fixation of parasite’s mouth area on the feeding site, while serrated setae of the mandibular palp and the squamose surface of the maxilla seem to be adapted for tissues rasping or scraping. Individually, the observed mouthparts in pulli do not give indication of the ”sucking” as a feeding strategy. However, Günther’s comparative morphological analysis of non-parasitic vs parasitic cymothoida [15] showed that in this parasitic genera the distal segments of mouth parts are tightly arranged around the mouth opening, forming a blunt cone, instead of a more expanded ”feeding pit” present in the non-parasites. This cone is formed by the labrum, labium and the distal maxillary part and secured with the maxillipede palps during the suction, which is exerted by the contractions of the oesophagus. The distal, freely movable parts of mandibles and maxillules are engaged in the attacking and destroying the skin of host animal.

Another cymothoiid, *Nerocila* sp. possesses mouthparts that form a distinct mouth cone used for true sucking, which is performed by their tight folding around each other, likewise providing functional rails for the movement of only mandible and maxillula [16]. Authors also recognised that mandible and maxillula seem to be able to cut off small tissue pieces from the host, facilitated by sharp incisor regions and spines of mandibles. Unlike *C. oestroides*, *Nerocila* sp. parasitises fish body surface, being likely more exposed to the environmental factors that consequently could have shaped a distinctive mouth apparatus enabling hemophagous feeding. Our longitudinal *C. oestroides* sections clearly show tissues fragments around isopod mouthparts and inside the mouth cavity, suggesting a mechanism of tissues grinding, rather than the blood sucking. *Cymothoa exigua*, in contrast is believed to extract blood through its frontal claws causing tongue atrophy, the latter being afterwards replaced by the isopod female [17]. Even though this species is also buccal cavity-dweller as *C. oestroides*, the extent of effected host tissues damage exceeds what we have observed in case of *C. oestroides*. This suggests that the feeding strategy even between closely related taxa could be very different. It is also noteworthy that Gunther’s concept of “sucking” mechanism in parasitic Cymothoida was illustrated on the examples of aegid genera *Aega* and *Rocinela* and cymothoidae genera *Cymothoa* and *Anilocra*, but not *Ceratothoa* itself [15].

As the mouthparts’ appearance cannot fully elucidate the exact feeding mechanisms, we have performed electron microscopy to assess whether the gut ultrastructure reveals physiological traits necessary for blood meal digestion. Namely, Romestand et al. [18] concluded that *C. oestroides* feeds cyclicly upon meal of sucked blood; before or at the start of the vitellogenesis, and after releasing of pulli, while blood absorption in gut seems to depend on parasite sexual and "intermoult" cycle. However, authors drew conclusions based on repletion of the mid-gut diverticula (syn. "hepatopancreatic caeca"), its colouring and weight during different sexual stages, missing to evidence the actual presence of blood in the ingesta or digesta. Moreover, only the attributed brownish colouring (from dark to light brown) of the passing digesta within diverticula was taken as a sign that the meal was composed of blood. The other factors influencing the general opinion of *C. oestroides* being a blood sucking parasite, include the existence of lateral oesophageal glands that we also observed but only in adult female, which seem to possess anticoagulative and antithrombic proprieties [19]; and the hemolytic activity of mid-gut diverticula [20]. The data available up to now do not conclusively suggest feeding upon blood, as we know that oesophageal glands or gut tissues produce and secrete/ excrete a myriad of products whose activity may not be exclusively related to blood meal digestion. In hematophageous insects, hemoglobin hydrolysis in digestive tract results in accumulation of prooxidant heme, whose immediate enzymatic degradation is prerequisite for the maintenance of physiological integrity of parasite tissues [21]. Heme oxygenase catalyses the oxidative degradation of heme to biliverdin, CO, and iron, pigmenting actually the digesta in green, not brown [21,22]. In addition, heme biocrystallisation observed in *Plasmodium* spp., *Rhodinus prolixus* (a vector of *Trypanosoma cruzi*), and trematodes *Schistosoma mansoni*, *Opisthorchis felineus*, and *Clonorchis sinensis* leads to the formation of hemozoin, large electron-dense and iron-rich crystalline aggregates present in gut after the blood meal [23,24,25]. We did not observe similar structures in adult female *C. oestroides*, which does not necessarily imply that the isopod potentially does not use other heme detoxifying mechanisms, still unknown. Namely, while hemozoin is produced among phylogenetically unrelated blood-feeders, Oliveira et al. [24] observed that the substance could not be identified in *Aedes* sp. and *Anopheles* sp. mosquitoes, nor in the hard tick (*Boophilus microplus*), suggesting that the acid environment during blood meal digestion is essential for its synthesis. Interestingly, while hemozoin synthesis has been observed in *O. felineus*, it is lacking in a closely related taxa *O. viverrini* [25].

The intestine of *C. oestroides* is thin-walled and composed of a monolayer of large epithelial cells with prominent nuclei. However, it does not seem to be lined with cuticle as seen in some other species of isopods [26] or at least it is not as prominent as in other species. Most of the cell nuclei appeared ovoid or round, with no or only small protrusions compared to nuclei seen in free-living isopod *Notatolana obtusata* [27]. Moreover, majority of cell nuclei were euchromatic, indicative of active gene expression. Presence of numerous vesicles and free ribosomes towards the apical portion of the cells as well as numerous small membrane invaginations of the basal cell membrane also supports high metabolic activity, even though the intestinal lumen was empty. Some of the cells had a large number of short filaments in their cytoplasm, indicating that these are in fact myocytes. However, it is hard to claim whether these cells are part of intestinal wall or sampling artefacts belonging to external musculature ensuring peristalsis and moving of digesta through the intestine. 

Apical membranes of hepatopancreatic cells are lined with densely packed microvilli, a feature shown to be present in other isopod species [27,28,29]. Large nuclei are ovoid in shape and lacking any projections as those seen *N. obtusata* [27] or amphibious isopod *Ligia italica* under starvation [30]. Although some cells appeared to have higher amount of heterochromatin, indicative of lower gene expression and by extension lower overall activity, it was shown that in *L. italica* part of hepatopancreatic cells were euchromatic, while others had higher amount of heterochromatin [29]. Moreover, numerous small vesicles and elaborate membrane invaginations indicate high cellular activity and trafficking of nutrients among cells, despite apparently higher heterochromatin content. Most of the cells contained several large lipid droplets, which is in accordance with findings in other isopod species [27,28,29]. However, contrary to these reports which found a certain amount of glycogen in hepatopancreatic cells cytoplasm, we evidenced no structures that would indicate glycogen storage, despite the granulated appearance of cytoplasm in some cells. The reason for this could be that the animals where in between two feeding cycles and, if present, glycogen was used by the time of sampling. While glycogen is essential substrate of anaerobic pathway of energy generation, lipids are degraded during oxidative metabolism [31]. Considering that *C. oestroides* lives in buccal cavity of its host with a sufficient oxygen supply where it can rely on oxidative metabolism, lipid storage over glycogen storage is more feasible, compared to some endoparasites, e.g. nematodes, in which glycogen storage is favored due to hypoxic environment of host’s body cavities in which they reside [32]. The presence of numerous vesicles around lipid droplets with likely proteinaceous content remains puzzling. It is unlikely that these are some artefacts considering their number, however more advanced sample preparation techniques or cytochemical techniques could reveal their true nature. However, when discussing the ultrastructure of intestine and especially hepatopancreas, it should be noted that in isopods the ultrastructure of these organs is dynamic and highly dependent on feeding cycle and environmental influences, as well as molting cycle [26]. Whether this is true for *C. oeastroides* as well remains unknown, but it can be speculated that this parasite does not undergo extensive starvation periods with the only limiting factor to the amount of food available being the rate to which the tongue epithelium of its host regenerates. 

Tissues reaction to *C. oestroides* confirms that the parasite does not provoke haemorrhaging at the feeding or attachment site. Marked hyperplasia of the squamous epithelium, with weak to moderate infiltration of inflammatory cells (macrophages-like cells, IgM^+^ cells, mast cells and eosinophil granulocytes) and decrease in mucous cells counts, supports a long-lasting and silenced inflammatory reaction. This is somewhat contrasting the usual more acute and proliferative response to the parasite infections in gills [33,34], but it seems that such interaction supports the maintenance of isopod with as minimal tissue damage as possible. As epidermal fish mucous contains a myriad of innate immune components [35], we can speculate that *C. oestroides* modulates the expression of mucous cells at the attachment/ feeding site so to avoid intense inflammation. Similarly, Atlantic salmon infected by low numbers of *Lepeophtheirus salmonis* showed strong reduction in mucous cell numbers in the skin at 24 h post-infection [36], while the branchurian *Argulus foliaceus* in the rainbow trout (*Oncorhynchus mykiss*) had no effect on mucous cell population of the fish head skin [37]. 

Further, positive labelling of Cox-2 in cells suggests a feeble infiltration of most likely macrophages colonising the oral mucosa. Cox-2 is an inducible inflammation-related enzyme which is responsible for the conversion of arachidonic acid into prostaglandins during the activation of innate immune response [38]. Modulation of *cox-2* gene expression has been studied for parasite and bacterial infections in many fish species, showing upregulation in rainbow trout (*Oncorhynchus mykiss*) infected by myxozoans *Myxobolus cerebralis* and *Tetracapsuloides bryosalmonae*, monogenean *Gyrodactylus derjavini*, and in sea bream (*Sparus aurata*) infected by *Photobacterium damselae* subsp. *piscicida* (see [38] for a review). To the best of our knowledge, only a single study previously assessed immunolabelling of cox-2 by this antibody, in the mummichog (*Fundulus hetereoclitus*), although in relation to salinity acclimation rather than response to infection [39]. This might indicate that cyclooxygenase 2 could also have a role in stress-related conditions in fish, in addition to early immune response to pathogens. Cox-2 increased expression contributes to the enhancement of inflammatory response during the initial phase of infections [38], which may justify why only a scarce number of Cox-2^+^ cells was present at the isopod attachment site. Namely, the isopod infection in collected specimens shows scarce evidence of granulocytes and macrophages infiltration, inferring a chronic inflammatory process, characterised mainly by tissues hyperplasia. 

The abundant proliferation of the epithelium highlights that the damage is limited only to the epithelium itself and does not involve its basement membrane, therefore the process is of regenerative rather than reparative nature. This was also evidenced by a strong reactivity of the mucosal cells to the PCNA antibody. Cell proliferation can be detected by the immunohistochemical labelling of the proliferating cell nuclear antigen (PCNA), which is an evolutionary highly conserved 36 kd protein, directly involved in DNA synthesis [40]. PCNA-positivity has been reported in organs of different fish, but scarce information exists regarding its expression in infected fish tissues [41]. Changes in PCNA expression can provide an early indication of alterations in normal epithelium functioning, as demonstrated in the brown trout (*Salmo trutta*) infected by the acanthocephalan *Dentitruncus truttae* [41]. Authors observed an increased number of PCNA-positive cells in both intestinal epithelium and submucosae layer in regions close to the point of parasite attachment. A similar need to repair the damage induced by parasite in the gill epithelium through hyperplasia and proliferation, although inferred at molecular level (RNAseq and qPCR), has been previously noted in infection by another Mediterranean widespread pathogen, *Sparicotyle chrysophrii* (Monogenea, Polyopisthocotylea) [42]. Authors also suggested that such enhanced process could represent an apoptotic stimulus, as uncontrolled proliferation correlates with a high level of apoptosis. In case of *C. oestroides*, it still remains to be tested whether apoptosis also plays a role in host-pathogen interaction. 

Expected infiltration of IgM^+^ cells (B lymphocytes, plasmablasts and plasma cells) at the infection site suggests the activation of a specific immune response to *C. oestroides*. Herein applied anti-European sea bass IgM was a polyclonal antibody specifically developed against sea bass IgM, previously used to label IgM^+^ cells in sea bass gills infected by *Amyloodinium ocellatum* [43]. Immunoglobulins secretion occurring locally in the mucosa is a consequence of antigen capture and phagocytosis by antigen-presenting cells, antigen presentation to T lymphocytes, and stimulation and differentiation of B-lymphocytes into plasmablasts and eventually plasma cells [44]. Both a local and systematic humoral response have been observed in *S. chrysophrii* infection, through a marked upregulation of genes involved in Ig construction (IgM heavy chain, Ig kappa chain V-I region Walker and Ig lambda-1 light cahin-like) [42], as well as in Atlantic salmon (*Salmo salar*) skin infected by salmon louse (*Lepeophtheirus salmonis*) [45]. 

However, observation of an unidentified bacterial biofilm on isopod mouthparts and ungulae prompt us to consider that IgM production could be alternatively a response to pathogen-associated molecular patterns (PAMPs). Ectoparasites can facilitate secondary bacterial infections mostly related to the mechanical damage of the mucosal surface at the attachment site, which constitute an entrance gate for opportunists. Parasites can also serve as vectors for pathogenic bacteria. Vibrios for example have a particular preference for chitinous surface of crustaceans to use as a substrate for colonization [46]. Members of the genus possess specific proteins and enzymes that can facilitate both attachment to chitinous surface and degradation of crustacean’s exoskeleton to obtain nutrients [46]. Aeromonads have also the same characteristics and the most notable species, *Aeromonas hydrophila* and *A. veronii* [47], are both important pathogens of European sea bass [48]. We do not know the species that epibiotic bacterial biofilm covering *C. oestroides* body parts belongs to, or whether it poses a potential threat to the fish host. On the other hand, epibiotic biofilms are quite common in solid outer surface of aquatic organisms, and their role is quite complex ranging from beneficial to detrimental for the host (the isopod herein). Microbial biofilms may also play a functional role taking part in metabolite exchanges and degradation of tissues [49]. In non-parasitic animals, epibiotic biofilm constitute a functional interface between host and environment, therefore following the same analogy, epibiotic biofilm of the parasite can act as a functional interface between parasite and its host. The role of epibiotic bacteria in ectoparasites, especially in relation to their interaction with parasitized host, is currently unknown and should be further studied.

## 4. Materials and Methods 

### 4.1. Fish Sampling

Fingerlings of European sea bass (*Dicentrarchus labrax*) (average length 14.5 cm ± 1.11 cm SD; average weight 33.2 g ± 5.67 g SD) were sampled from a commercial aquaculture facility during a vaccination campaign. Buccal cavity was checked for presence of *Ceratothoa oestroides* and infected (n = 20) and uninfected (n = 10) fish were euthanised by anaesthetic overdose (MS-222, 0.1 g/l), and the head was separated from the body for downstream analyses. 

### 4.2. Histology and Immunohistochemistry

European sea bass (*Dicentrarchus labrax*) heads (5 infected and 5 uninfected) were immediately fixed in Bouin’s fixative for 24 h at 4 °C and subsequently washed 3 times in 70% EtOH, decalcified for 1 h and then embedded in paraffin to obtain sagittal sections, using a standard histological protocol. Serial sections of 4 μm were stained with haematoxylin-eosin or submitted to immunohistochemical protocol (IHC). 

To localise particular inflammation- and proliferation-related antigens at the *C. oestroides* parasitation site, serial histological sections from infected and uninfected fish were subjected to IHC using HRP-based kit (EnVisionTM FLEX, Dako, Agilent). Briefly, slides were routinely deparaffinised and rehydrated (room temperature; RT, in a humid chamber), tissue endogenous peroxidases were inactivated by immersion in 3% H_2_O_2_ (30 min, RT), and an antigen retrieval treatment (10 min at 90 °C) was performed using high pH Target Retrieval Solution (TRS) buffer, or alternatively low pH TRS buffer (Dako, Agilent). Unspecific antibodies binding was blocked with 1:20 normal goat serum (30 min, RT). 

Subsequently, primary antibodies were incubated for 2 h at RT: rabbit polyclonal anti-European sea bass immunoglobulin M (anti-ESB IgM, dilution 1:24,000) (Trieste University, Italy), rabbit polyclonal anti-inducible nitric oxide synthase (iNOS, dilution 1:200) (RB-1605, Thermo Scientific); mouse monoclonal anti-proliferating cell nuclear antigen (anti-PCNA, dilution 1:50) (SC-56, Santa Cruz Biotechnologies), rabbit polyclonal anti-cytokeratin (Cytokeratin, dilution 1:50) (ab9377, Abcam); rabbit polyclonal anti-C reactive protein (CRP, dilution 1:150) (CRP-16A, LiStarFish, Alpha Diagnostics), mouse polyclonal anti-cyclooxygenase 2 (Cox-2, dilution 1:50) (160126, Cayman Chemicals, CABRU sas), and rabbit polyclonal anti-cluster of differentiation 83 (anti-CD83, dilution 1:30) (HPA041454, Sigma Life Science). Slides were incubated with EnVision anti-mouse- or anti-rabbit-linkers (15 min, RT), EnVision HRP reagent (20 min, RT), diaminobenzidine (Sigma Aldrich) as a chromogen (7 min) and haematoxylin (1 min) as a counterstain. In negative control sections, primary antibodies were replaced by dilution buffer. Slides were observed under the light microscope (Leica DMRB) equipped with digital camera (Nikon) and imaging software (NIS-Elements Br, Nikon). 

### 4.3. Micro-computational Tomography (µ-CT) 

Infected fish samples (n = 5) were fixed in buffered 4% formaldehyde as for routine histology, subsequently dehydrated to 70% ethanol for 3 days before scanning performed at the Hellenic Centre for Marine Research (HCMR). Phosphotungstic acid (PTA, 0.3% v/v) in 70% ethanol was used to enhance contrast among the soft tissues (Metscher 2009). The µ-CT (SkyScan 1172 micro-CT scanner; SkyScan, Bruker, Belgium) uses a tungsten X-ray source with an anode voltage ranging from 20 to 100 kV, 11 PM CCD camera (4000 × 2672 pixel) and a maximal resolution of < 0.8 μm/pixel. Adult males and females were scanned at a voltage of 59 kV and 167 μA with an aluminium filter for a full rotation of 360° at the highest camera resolution. The projection images acquired during the scanning procedure, were reconstructed into cross section images using the SkyScan’s NRecon software (NRecon, Skyscan, Bruker, Belgium) equipped with a modified Feldkamp’s back-projection algorithm. 3D volume renderings of the scanned specimen were created using the CTVox software (CTVox, Skyscan, Bruker, Belgium) to visualise the anatomy of the parasite. 

### 4.4. Descriptions of C. oestroides Intramarsupial Embryonic Stages and Mouthparts

Head appendages of intramarsupial stages (n = 5) were removed using dissecting needles and micro forceps under the stereomicroscope (WILD). Temporary mounts of mouthparts were prepared in lactic acid (min. 88% C_3_H_6_O_3_). All drawings were made using OLYMPUS BH2 optical microscope equipped with a drawing tube (camera lucida), using magnifications of 100x for intramarsupial embryonic stages, and 200x and 400x for mouthparts. Descriptions were prepared using descriptive terminology according to Backenhaster et al. [50] for embryonic stages, and Hadfield et al. [51] for mouthparts.

### 4.5. Scanning Electron Microscopy (SEM)

Specimens (n = 5) were fixed in 2% buffered glutaraldehyde, washed in sodium cacodylate buffer, post-fixed with 1% OsO_4_ and dehydrated in an ascending alcohol series. Specimens mounted on stubs were gold palladium sputter-coated and examined using JEOL JSM-6390LV operating at 15kV (Electron Microscopy Laboratory of the University of Crete).

### 4.6. Transmission Electron Microscopy (TEM)

Intestines and hepatopancreases were removed from adult females (n = 5) under a stereomicroscope Olympus SZX10 using dissecting needles and micro forceps. Samples were immediately fixed in ice-cold 4% paraformaldehyde. Afterwards, samples were washed in phosphate buffered saline (PBS) (3x, 15 min each) and post-fixed in 1% aqueous osmium tetroxide for 2 h at room temperature. In-block staining with 2% aqueous uranyl acetate was performed overnight at room temperature, followed by washing in deionized water, dehydration in an ascending series of acetone (30–100%) and embedding in Durcupan resin (Honywell-Fluka, US). Semi-thin sections were cut to 0.5 μm, stained with 1% toluidine blue and inspected under a light microscope for orientation. Ultrathin sections were cut to 0.06-0.07 μm, placed on 100-mesh copper grids, stained with uranyl acetate and lead citrate according to Reynolds [52] and inspected under Jeol 1010 (Jeol, Japan) TEM operating at an accelerating voltage of 80 kV. Images were captured with a Mega View III camera (Olympus Soft Images Solutions GmBH, Germany) and assembled and annotated in Photoshop CS5 software (Adobe Systems, US). 

### 4.7. Animal Ethics

Fish were sampled during procedural health examination at the aquaculture facility performed by a veterinary service, and in accordance to the Guidelines of the European Union Council (Directive 2010/63/EU) and the Croatian legislation (Zakon o zaštiti životinja; NN 135/06 and 37/13, Pravilnik o zaštiti životinja koje se koriste u znanstvene svrhe; NN 55/13). The procedures have been approved by the Committee for the Animal Welfare, Institute of Oceanography and Fisheries, Split, Croatia (IACUC, approval number 134/2). All efforts were made to minimize suffering of the fish used for the study in accordance to aforementioned national legislation and the current European Union legislation (86/609/EU).
